# Birdsong classification based on ensemble multi-scale convolutional neural network

**DOI:** 10.1038/s41598-022-12121-8

**Published:** 2022-05-23

**Authors:** Jiang Liu, Yan Zhang, Danjv Lv, Jing Lu, Shanshan Xie, Jiali Zi, Yue Yin, Haifeng Xu

**Affiliations:** 1 College of Big Data and Intelligent Engineering, Southwest Forestry University, Kunming, 650000 China; 2 College of Mathematics and Physics, Southwest Forestry University, Kunming, 650000 China; 3 School of Information Science and Technology, Beijing Forestry University, Beijing, 100091 China

**Keywords:** Machine learning, Computer science, Information technology

## Abstract

With the intensification of ecosystem damage, birds have become the symbolic species of the ecosystem. Ornithology with interdisciplinary technical research plays a great significance for protecting birds and evaluating ecosystem quality. Deep learning shows great progress for birdsongs recognition. However, as the number of network layers increases in traditional CNN, semantic information gradually becomes richer and detailed information disappears. Secondly, the global information carried by the entire input may be lost in convolution, pooling, or other operations, and these problems will weaken the performance of classification. In order to solve such problems, based on the feature spectrogram from the wavelet transform for the birdsongs, this paper explored the multi-scale convolution neural network (MSCNN) and proposed an ensemble multi-scale convolution neural network (EMSCNN) classification framework. The experiments compared the MSCNN and EMSCNN models with other CNN models including LeNet, VGG16, ResNet101, MobileNetV2, EfficientNetB7, Darknet53 and SPP-net. The results showed that the MSCNN model achieved an accuracy of 89.61%, and EMSCNN achieved an accuracy of 91.49%. In the experiments on the recognition of 30 species of birds, our models effectively improved the classification effect with high stability and efficiency, indicating that the models have better generalization ability and are suitable for birdsongs species recognition. It provides methodological and technical scheme reference for bird classification research.

## Introduction

As the construction of ecological civilization advances, methods for efficient and quick assessment of the quality of the environment need to be further studied. Birds play an essential role in the ecosystem, and their communities are a crucial indicator of environmental quality^1,2^. The study of birds is of great significance for protecting birds, understanding wetland ecosystems and evaluating the quality of ecosystems. The International Union for Conservation of Nature (IUCN 2014)^3^ listed that there are 1373 bird species in the world and more than 13% of species are vulnerable and even face immediate danger of extinction. Due to the characteristics of birds with high flexibility of movement, extensive moving range, and strong environmental adaptability, birdsong, a sign of activities of birds, is often used to detect, monitor, and quantify species. Birdsong contains rapid time modulation, and has stability in the same species and discrimination between species. Automatic bird classification model, established with birdsong audio data, has many potential applications in protection, ecology and archives^4^.

Research on birdsongs has demonstrated that human language and birdsongs have striking analogies in vocal articulation and neural functionality^5^. Therefore, many researchers in bird song recognition often use MFCC as extracted audio features. In addition, to better analyze bird song, audio data is usually converted into a spectrogram with methods such as short-time Fourier transform (STFT)^6,7^ and wavelet^8,9^. Many researchers have carried out a lot of research based on traditional machine learning methods for birdsong classification. For limited data, an automated birdsong phrase classification algorithm, dynamic time warping (DTW), is developed to reduce the need for manual annotation^10^. Ladislav Ptacek^11^ and Chang-Hsing Lee^12^ using Gaussian Mixture Model (GMM) to classify birds on different feature data sets, have achieved good results. Douwe Gelling^13^ used HMM and GMM models to study the importance of using time information in recognizing bird vocalizations. Diego Rafael Lucio^14^ built a support vector machines (SVM) classification model based on the acoustic and visual features extracted from birdsong, and obtained an accuracy rate of 91.08%.

In recent years, deep learning^15^, which learns feature representation via a hierarchical structure, has achieved remarkable success in various fields. Inspired by this, Ahmad Salman^16^ used the deep learning method in the LifeCLEF14 and LifeCLEF15 fish data sets to achieve a classification rate of more than 90%. Le-Qing Zhu^17^ proposed a cascade structure combined deep convolutional neural networks (DCNNs) and SVM to identify lepidopteran insects by images. In addition, deep learning is also widely used in birdsong research. Piczak^18^ and Tóth^19^ in the BirdCLEF 2016 competition, used deep learning to identify birdsong with good results. Gaurav Gupta^20^ presented a deep learning approach targeting large-scale prediction and analyzed bird acoustics from 100 different species. Xie^21^ used selectively fuse model on classifying 43 bird species and increased the classification performance effectively. Although, semantic information becomes richer, detailed information disappears as the number of layers in the network increases^33^. In addition, the global information carried by the entire input may be lost in convolution, pooling, or other operations, which may affect the performance of the classification^34^. To mitigate this problem, Di Wang^22^ proposed a multi-scale information compensation module on CNN. By integrating the original input with more abstract hierarchical learning feature maps, this module maintained detailed semantic information. Research has demonstrated multi-scale is suitable for computing hierarchical features and successful in a range of pixel-level prediction tasks^23–26^. It can be seen that deep learning models and Multi-scale CNN models have powerful classification capabilities and can be used in a variety of research fields.

Ensemble learning is well known effective method for combining multiple learning methods to yield better performance^27^. Ensemble methods have been applied in many research fields such as computational intelligence, statistics, and machine learning^28^. Zhao^29^ reported the application of ensemble neural networks. Compared with a single neural network model, an ensemble neural network can effectively improve the generalization ability of the classifier. Antipov^30^ proposed a convolutional neural network ensemble model to improve the state-of-the-art accuracy of gender recognition from face images on one of the most challenging face image datasets. In summary, ensemble learning has carried out a lot of research in different fields, which provides theoretical support for the follow-up research of this paper.

Therefore, classify birds through bird songs based on modern computer technology greatly promote ecological, environmental protection, and biodiversity research. To improve the performance and the knowledge gained from it, we adopted deep learning, transfer learning and ensemble technology on spectrogram in this paper. Our work proposed a multi-scale deep learning model and an ensembled multi-scale deep learning model, characterized by constructing classification models using wavelet spectrogram of birdsong. The contributions of the current work are: (1) The wavelet spectrograms of 30 kinds of bird songs indicate good separability; (2) We propose a multi-scale convolution kernel decomposition method, which can effectively generate multiple convolution kernels from a fixed scale; (3) A multi-scale CNN(MSCNN) model and an ensembled multi-scale CNN(EMSCNN) model are constructed for the different convolution kernels. Our models achieve a better performance than LeNet, VGG16, MobileNetV2, ResNet101, EfficientNetB7, Darknet53 and SPP-net models.

This paper is organized as follows: Firstly, we describe the proposed approach for birdsong recognition, which mainly includes wavelet spectrogram generation, multi-scale CNN model and ensemble multi-scale CNN model construction. Secondly, describe the experiment design. Thirdly, discuss and analyze the experimental results. Finally, present conclusions and directions for future work.

## Materials and methods

### Wavelet spectrogram

The wavelet transform (WT) is the typical time–frequency analysis method^31^. It combines the characteristics of time-domain and frequency-domain. The features on the wavelet scale can also analyze the changes of frequency components over time. Wavelet transform uses a finite-length or fast-decaying "mother wavelet" oscillating waveform to represent a signal; the "mother wavelet" is multi-scaled and translated to match the input signal. The WT provides a time–frequency window that can be modulated, and the width of the window changes with frequency, which make it more suitable for non-stationary signal analysis.

The wavelet transforms, $W_{f} \left( {\alpha ,\beta } \right)$, of a time signal $s\left( t \right)$ is given by:

1 $${W_{f} \left( {\alpha ,\beta } \right) = \int\limits_{{ - \infty }}^{{ + \infty }} {s(t)\psi _{{\alpha ,\beta }}^{*} \left( t \right)dt = \frac{1}{{\sqrt \alpha }}\int\limits_{{ - \infty }}^{{ + \infty }} {s(t)f^{*} \left( {\frac{{t - \beta }}{\alpha }} \right)dt} } }$$

where $\psi^{*}_{\alpha ,\beta } \left( t \right)$ is the complex conjugate of $\psi_{\alpha ,\beta } \left( t \right)$ shown in formula (2).

2 $$\begin{array}{*{20}c} {\psi_{\alpha ,\beta } \left( t \right) = \frac{1}{\sqrt \alpha }f\left( {\frac{t - \beta }{\alpha }} \right) } \\ \end{array}$$

where $\psi_{\alpha ,\beta } \left( t \right)$ scans and translates the signal $s\left( t \right)$ to wavelet domain, where $\alpha$ is the dilation parameter for changing the oscillating frequency and $\beta$ is the translation parameter. The basis function for the wavelet transform is given in terms of translation parameter $\beta$ and dilation parameter $\alpha$ with the mother wavelet represented as:

Morlet wavelets have been found to be the most responsive wavelets to birdsongs^35^. The complex morlet wavelet is defined by formula (3) in the time domain:

3 $$\begin{array}{*{20}c} {\psi_{Morlet} \left( t \right) = \frac{1}{{\sqrt {\pi f_{b} } }} \cdot e^{{j2\pi f_{c} t - \left( {t^{2} /f_{b} } \right)}} } \\ \end{array}$$

where $f_{c}$ is the center frequency and $f_{b}$ is the bandwidth.

**Figure 1:**
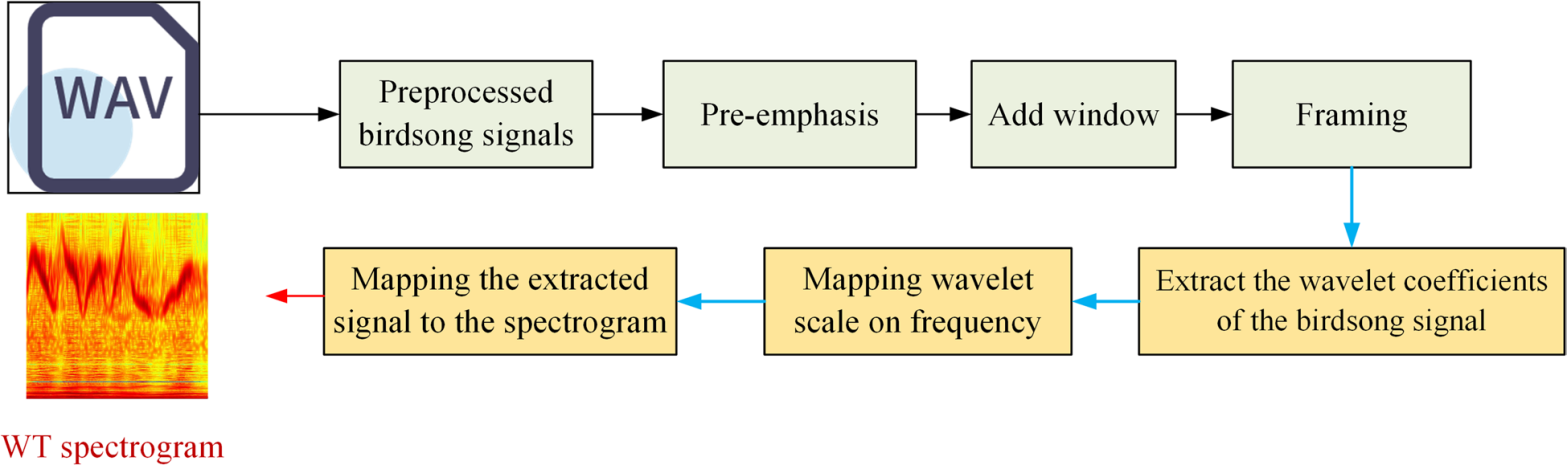
The process of wavelet spectrogram generation.

Based on the good characteristics of the wavelet transform, this paper chooses wavelet transform to generate birdsong spectrograms. The process of the wavelet spectrogram is shown in Fig. 1. Firstly, pre-emphasis and add window for birdsongs audio. Then the input audio signal wavelet coefficients are extracted, and the wavelet scale is mapped to the frequency domain. Finally, the extracted signal is mapped to the spectrogram.

### Multi-scale CNN classification model construction

Convolutional Neural Network (CNN) is a feedforward neural network, a representative algorithm of deep learning, with convolution calculation and deep structure^32^. CNN comprises input, convolution, pooling, fully connected and output layers. Generally speaking, CNN uses convolution to simulate feature extracted, reduces network parameters through weight sharing, reduces network dimensions through pooling, and finally completes the classification task through a fully connected network. The traditional convolution process can be defined as follows:

4 $$\begin{array}{*{20}c} {S = Conv\mathop \sum \limits_{k = 1}^{n} X_{{\left( {i,j} \right)}} *W_{k} + b_{k} } \\ \end{array}$$

where $i, j$ are the abscissa and ordinate of the image input, $n$ is the number of convolution operations, $X$ is the input image feature matrix, $W_{k}$ is the weight matrix of the convolution kernel $k$, $b_{k}$ is the bias, and $S$ is the result of feature matrix, $*$ represents the convolution operation.

**Figure 2:**
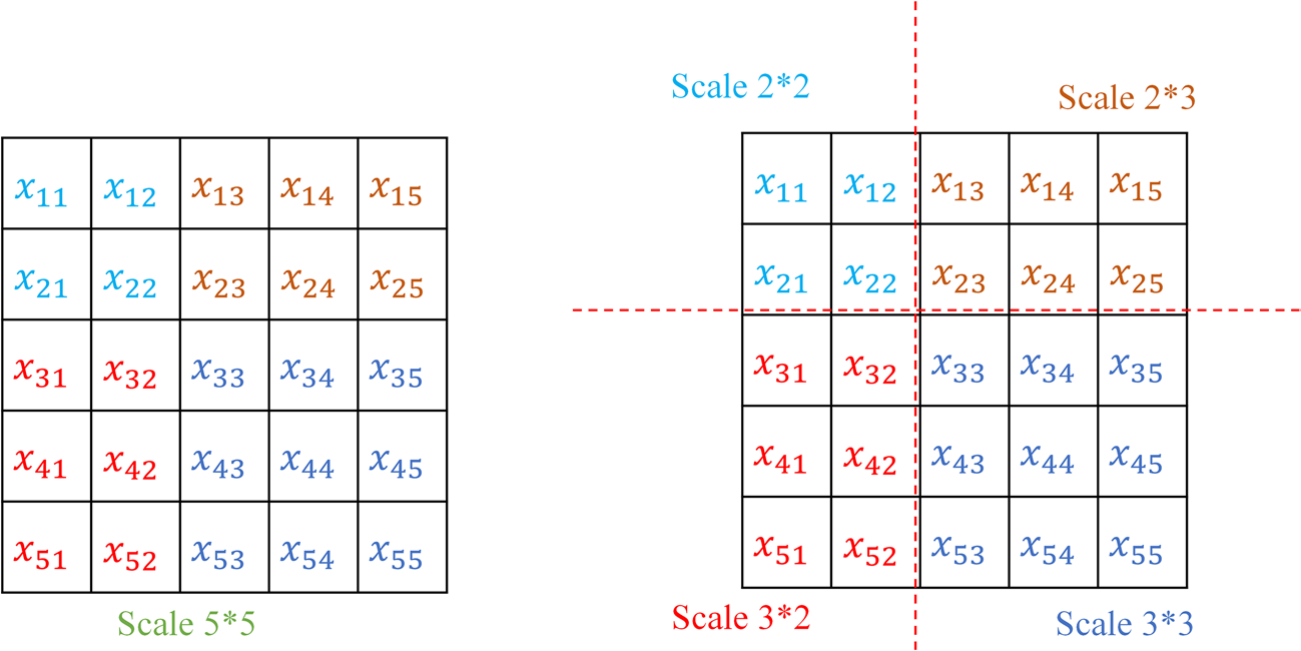
The 5*5 convolution kernel decomposition diagram.

Feature extraction is affected by the scale of the convolution kernel. Sometimes a single convolution kernel cannot fully extract the key features in a complex image, resulting in the loss of some key features. So, a new multi-scale method is proposed, which uses multiple convolution kernels to obtain features at multiple scales. In this paper, the multiple different scale convolution kernels are derived from the decomposition of large-scale. Taking the 5 × 5 convolution kernel as an example, its decomposed process is shown in Fig. 2, and the multi-scale convolution kernel is shown in formula 5.

5 $${Scale_{5*5} \mathop{\longrightarrow}\limits^{decomposition}\left[ {Scale_{2*2} ,Scale_{2*3} ,Scale_{3*2} ,Scale_{3*3} } \right]}$$

In the constructed CNN model, each convolutional layer uses a different convolution kernel to form a multi-scale convolutional neural network (MSCNN). The operation is defined as:

6 $$\begin{array}{*{20}c} {MSCNN = Conv\mathop \sum \limits_{m = 1}^{p} X_{{\left( {i,j} \right)}} *W_{{q_{m} }} + b_{{q_{m} }} } \\ \end{array}$$

where $q_{m }$ is the set of the multiple scales, such as $\left[ {Scale_{2*2} ,Scale_{2*3} ,Scale_{3*2} ,Scale_{3*3} } \right]$, $W_{{q_{m} }}$ is the weight matrix of the convolution kernel $q_{m}$ and $b_{{q_{m} }}$ is bias, $p$ represents the number of layers in the network.

**Figure 3:**
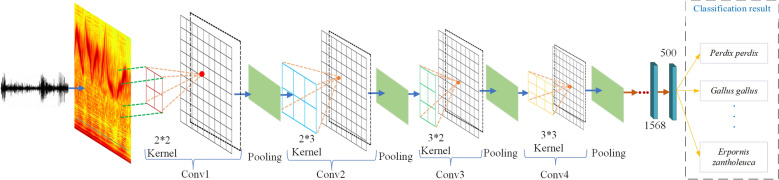
MSCNN model structure.

The MSCNN for birdsong classification model is established as shown in Fig. 3 based on the set of the multiple scales kernel in Fig. 2.

In CNN, the size of the convolution kernel determines the final learned features. The spectral image has the nature of high-dimensional features, which makes it challenging to apply a single convolution kernel. Using a deep CNN network can obtain more information by extracting deeper features, but many useful features will lose as the number of model layers increases. Eventually, the recognition of complex small samples of high-dimensional images becomes difficult. To increase the richness and diversity of model, this work ensembles different scale CNN to achieve better performance. The ensemble multi-scale convolutional neural network (EMSCNN) is defined as:

7 $$EMSCNN = SoftMax\left\{ {Concatenate\left( {Pooling\left\{ {Conv\left( {a_{1} *\left( {\begin{array}{*{20}c} {x_{11} } & {x_{12} } \\ {x_{21} } & {x_{22} } \\ \end{array} } \right)} \right)} \right\}\;Pooling\left\{ {Conv\left( {a_{2} *\left( {\begin{array}{*{20}c} {x_{13} } & {x_{14} } & {x_{15} } \\ {x_{23} } & {x_{24} } & {x_{25} } \\ \end{array} } \right)} \right)} \right\},\;Pooling\left\{ {Conv\left( {a_{3} *\left( {\begin{array}{*{20}c} {x_{31} } & {x_{32} } \\ {x_{41} } & {x_{42} } \\ {x_{51} } & {x_{52} } \\ \end{array} } \right)} \right)} \right\},\;Pooling\left\{ {Conv\left( {a_{4} *\left( {\begin{array}{*{20}c} {x_{33} } & {x_{34} } & {x_{35} } \\ {x_{43} } & {x_{44} } & {x_{45} } \\ {x_{53} } & {x_{54} } & {x_{55} } \\ \end{array} } \right)} \right)} \right\}} \right)} \right\}$$

**Figure 4:**
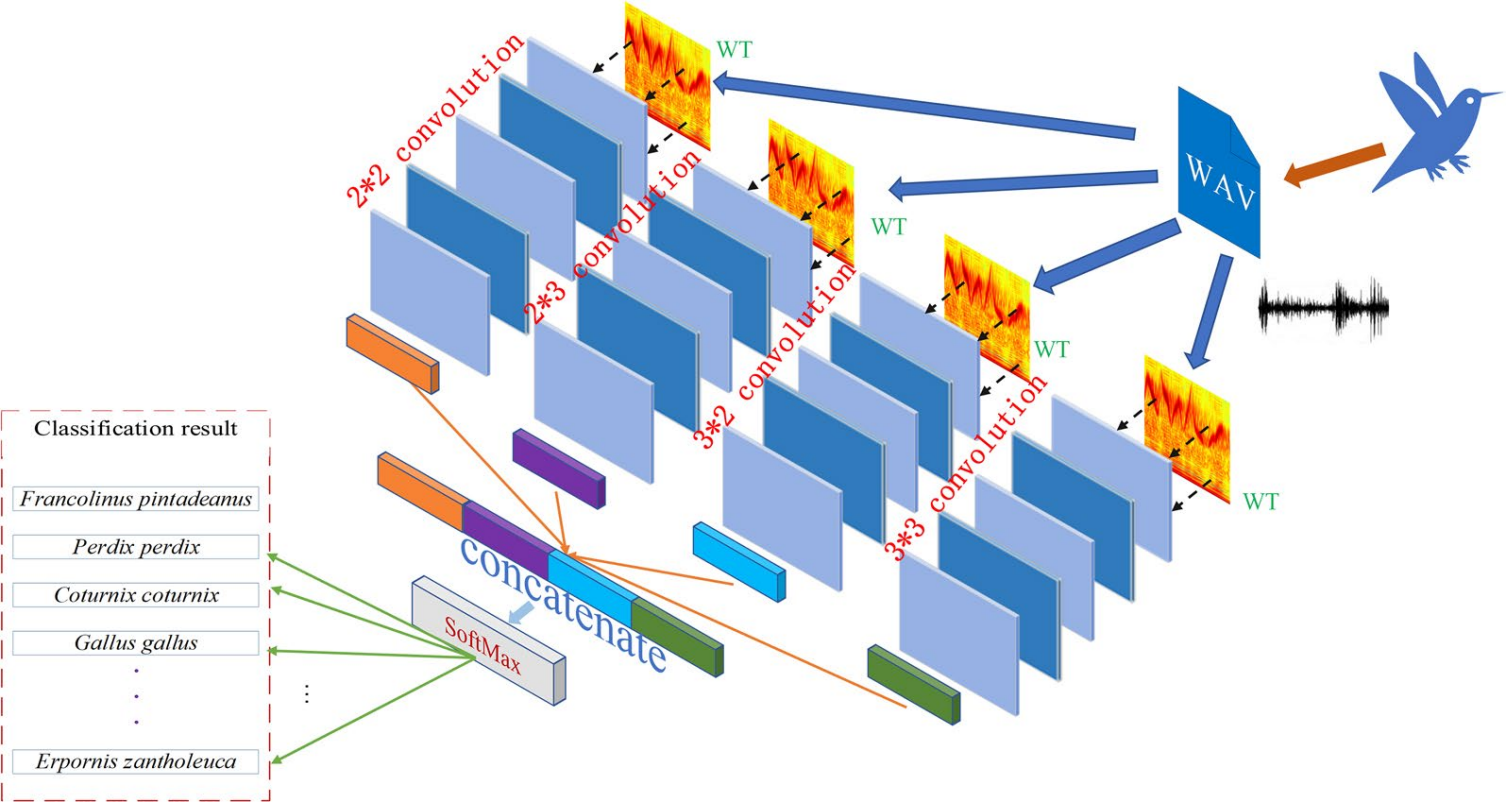
EMSCNN model structure.


Here, we use the fusion method to ensemble the calculation results under different convolution kernel scales. In formula (7) $a_{i}$ represents data vector and convolutes with the muti-scale convolution kernel. The pooling intermediate results of different scale CNN models are connected through the concatenate method, and then the classification results are output through SoftMax. The structure of birdsong classification model based on EMSCNN is shown in Fig. 4. How to train the EMSCNN model is described in Procedure 1. After the model is trained, we can use it to classify birdsong.

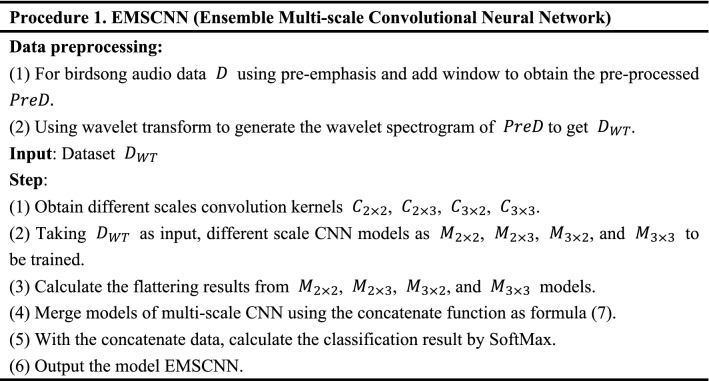


### Model evaluation

The performance of our proposed birdsong classification model was evaluated using accuracy, precision, recall, F1-score, Top-1, and Top-5. Among them, TP is genuinely positive, indicating the number of samples that have been correctly classified as actual samples. TN is a true negative, which means that the number is correctly classified as not accurate. FP is false positive, which means the number of falsely classified samples as actual samples. FN is a false negative representing the number of actual labels that the classification model did not predict.

Accuracy: Represents the percentage of correct predictions.

8 $$\begin{array}{*{20}c} {Accuracy = \frac{TP + TN}{{TP + TN + FP + FN}}} \\ \end{array}$$

Precision: Indicates the proportion of samples with correct predictions among samples whose actual values are positive.

9 $$\begin{array}{*{20}c} {Precision = \frac{TP}{{TP + FP}}} \\ \end{array}$$

Recall: Indicates the proportion of samples whose actual values are positive and predicted to be positive.

10 $$\begin{array}{*{20}c} {Recall = \frac{TP}{{TP + FN}}} \\ \end{array}$$

F1-score: Takes into account the precision and recall of the classification model.

11 $$\begin{array}{*{20}c} {F_{1} - score = \frac{2*precision*recall}{{precision + recall}}} \\ \end{array}$$

Top-1: It means that the largest probability vector among the predicted results is taken as the expected result. If the classification with the most considerable probability in your predicted effect is correct, the prediction is accurate. Otherwise, the prediction is wrong.

Top-5: Among the results of the classification model prediction, the top five with the largest probability vector are selected. As long as the correct probability appears in the top five, the prediction is accurate. Otherwise, the prediction is wrong.

### Experimental platform

The hardware platform used in this experiment is a desktop computer with 128G memory, Ryzen 9 5950X with 16 core and 32 thread CPU, 3.40 GHz frequency and 3090 24G GPU. The operating system is Windows 10 64-bit professional operating system. Annaconda3, PyCharm 2020.1, Python 3.7, TensorFlow 2.6.0 as deep learning platform and MATLAB 2018 as data processing platform are exploited.

### Ethics declarations

In this paper, the experiments did not use live birds.

## Experimental designs

**Figure 5:**
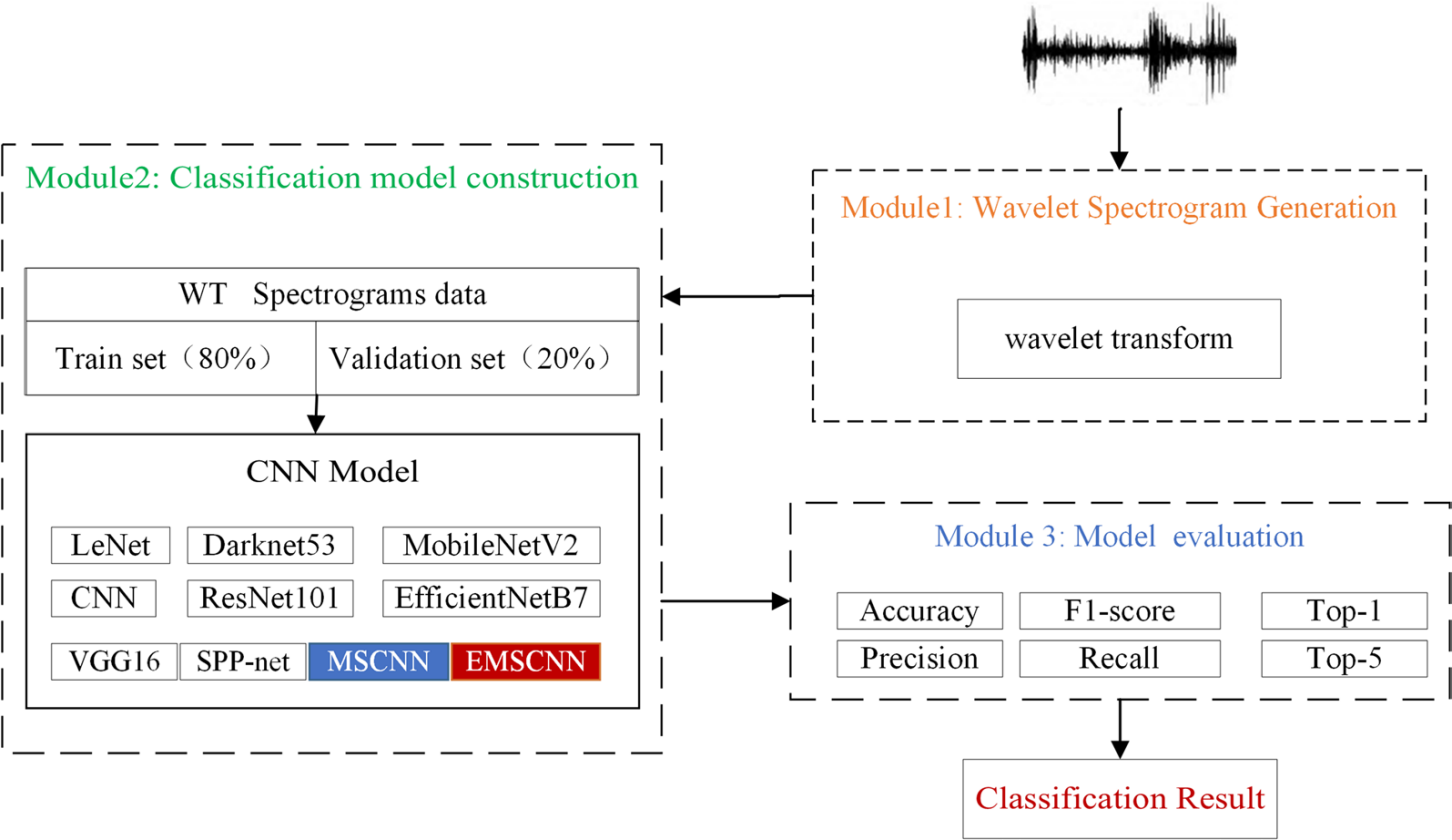
Experimental design process.

The experiments contain three modules: wavelet spectrogram generation, classification model construction and evaluation. Firstly, wavelet spectrograms are generated for collecting bird song data. Then, the MSCNN and EMSCNN models are built on WT spectrograms, and finally, the classification results are obtained through SoftMax. The detail of the experimental design process is shown in Fig. 5.

Module 1: Wavelet spectrogram generation. Wavelet spectrogram is generated using wavelet transform for collecting bird song data.

Module 2: Classification model construction. The spectrogram of birdsongs is divided into training and validation sets in the ratio of 8:2. The MSCNN and EMSCNN are built by training with input WT spectrograms and compared with the state-art such as LeNet, VGG16, MobileNetV2, ResNet101, EfficientNetB7, Darknet53, and CNN.

Module 3: Classification model evaluations. The indicators, Accuracy, Precision, F1-score, Recall, Top-1 and Top-5, are adopted to evaluate the performance of the above classification models.

## Results and discussion

### Wavelet spectrogram of birdsongs

In this study, a total of 30 kinds of birdsong data were collected from the public birdsong dataset (https://www.xeno-canto.org/ and http://www.birder.cn/). Table 1 lists information of 30 species of birdsongs including Latin name, genus, family name, and the number of wavelet spectrogram samples of birdsongs for each specie.

**Table 1:** Description of dataset.

Number	Latin name	Genus	Family	Spectrogram samples
1	*Francolinus pintadeanus*	*Francolinus*	*Phasianidae*	690
2	*Perdix perdix*	*Perdix*	*Phasianidae*	1140
3	*Coturnix coturnix*	*Coturnix*	*Phasianidae*	1367
4	*Gallus gallus*	*Gallus*	*Phasianidae*	1024
5	*Phasianus colchicus*	*Phasianus*	*Phasianidae*	1001
6	*Lagopus muta*	*Lagopus*	*Phasianidae*	973
7	*Lyrurus tetrix*	*Lyrurus*	*Phasianidae*	1180
8	*Cygnus olor*	*Cygnus*	*Anatidae*	1444
9	*Cygnus cygnus*	*Cygnus*	*Anatidae*	1135
10	*Branta canadensis*	*Branta*	*Anatidae*	580
11	*Anas platyrhynchos*	*Anas*	*Anatidae*	594
12	*Aythya fuligula*	*Aythya*	*Anatidae*	800
13	*Asio otus*	*Asio*	*Strigidae*	696
14	*Asio flammeus*	*Asio*	*Strigidae*	1900
15	*Grus grus*	*Grus*	*Gruidae*	758
16	*Numenius phaeopus*	*Numenius*	*Scolopacidae*	1802
17	*Glareola maldivarum*	*Glareola*	*Glareolidae*	956
18	*Larus canus*	*Larus*	*Laridae*	692
19	*Milvus migrans*	*Milvus*	*Accipitridae*	1668
20	*Haliaeetus albicilla*	*Haliaeetus*	*Accipitridae*	812
21	*Accipiter nisus*	*Accipiter*	*Accipitridae*	1066
22	*Accipiter gentilis*	*Accipiter*	*Accipitridae*	647
23	*Falco tinnunculus*	*Falco*	*Falconidae*	803
24	*Cettia cetti*	*Cettia*	*Sylviidae*	1500
25	*Acrocephalus arundinaceus*	*Acrocephalus*	*Sylviidae*	2934
26	*Phylloscopus trochiloides*	*Phylloscopus*	*Sylviidae*	1022
27	*Phylloscopus plumbeitarsus*	*Phylloscopus*	*Sylviidae*	1236
28	*Elachura formosa*	*Spelaeornis*	*Sylviidae*	848
29	*Leiothrix lutea (Scopoli)*	*Leiothrix*	*Sylviidae*	754
30	*Erpornis zantholeuca*	*Yuhina nigrimenta*	*Sylviidae*	800

**Figure 6:**
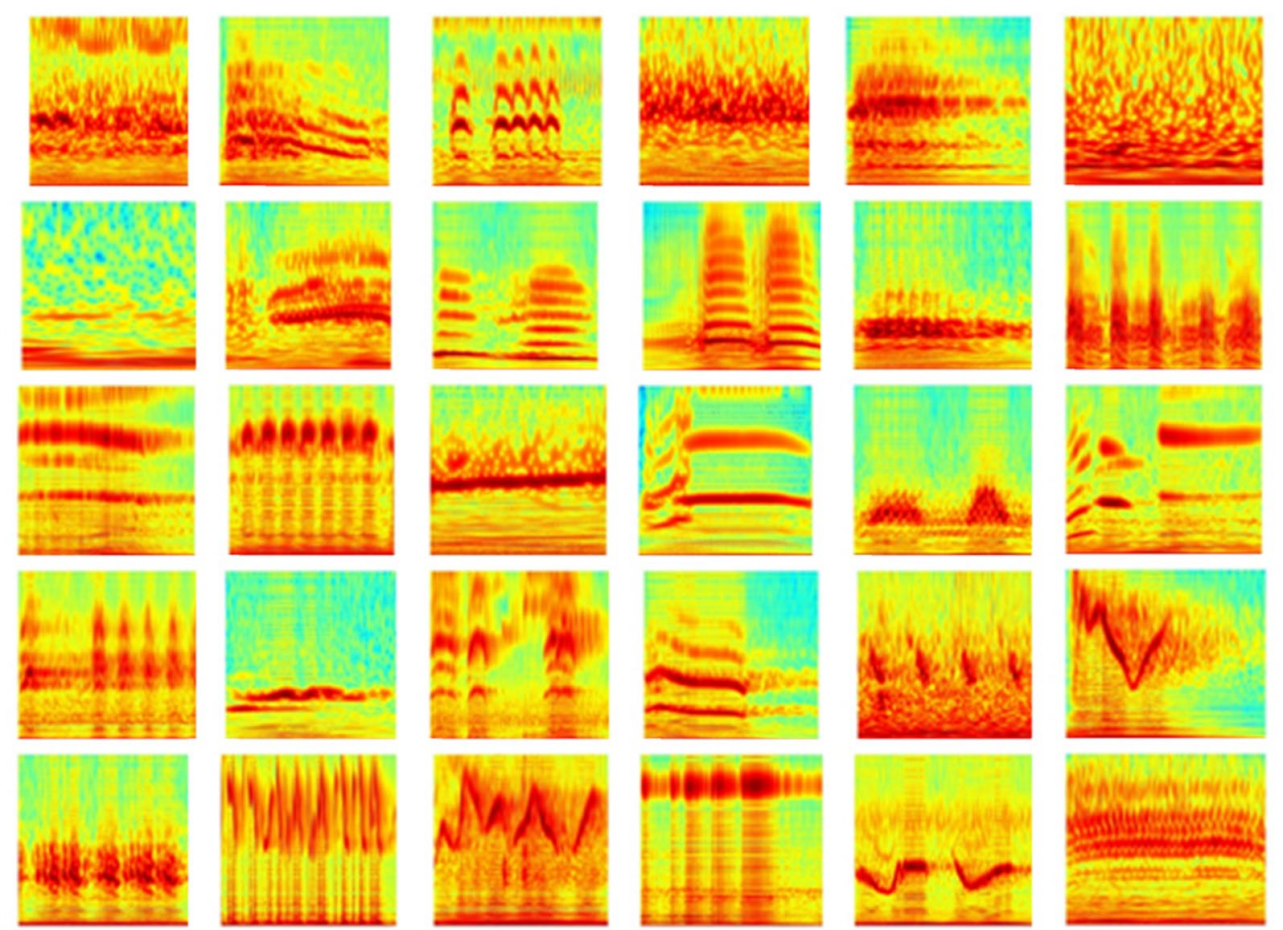
Wavelet spectrograms. The WT spectrogram is arranged from top to bottom and from left to right according to the bird number in Table1. The x-axis and y-axis of the wavelet spectrogram represents the time domain and frequency-scale domain respectively, and the color is energy information, the hotter color the more energy is.

The wavelet spectrograms of 30 species of birdsongs are shown in Fig. 6. From the wavelet spectrograms of birdsongs, we can clearly see that there are great differences between different species of birdsongs. The results show that the use of wavelet spectrograms to classify birds has practical significance.

### Experimental results

The experiment constructed the following models: LeNet, VGG16, MobileNetV2, ResNet101, EfficientNetB7, Darknet53, SPP-net, CNN Scale 2 × 2 (CNN-S22), CNN Scale 2 × 3 (CNN-S23), CNN Scale 3 × 2 (CNN-S32), CNN Scale 3 × 3 (CNN-S33), CNN Scale 5 × 5 (CNN-S55), our MSCNN and EMSCNN. Epoch is set to 30 times; the optimization function is Adam. The activation function used by the convolutional layer of the CNN model is ReLU. The evaluation of the above models is completed through the above 6 indicators.

In this work, CNN architecture with different scales is presented as ‘CNN-SXX’, where ‘XX’ stands for the kernel size. For example, CNN-S23 refers to the kernel size is 2 × 3. The structure of CNN models is the same except for the different scales of the convolution kernel. The CNN parameters are listed in Table 2, and the kernel Size of CNN-SXX models are listed in Table 3.

**Table 2:** CNN model structure parameters.

Layer	Name	Type	Kernel size	Stride	Filters	Input Size
1	Conv Input	Input Layer	–	–	–	112 × 112 × 3
2	Conv 1	Convolution2D	n × m	1	64	112 × 112 × 3
3	Pool 1	MaxPool2D	2 × 2	2	–	112 × 112 × 64
4	Conv 2	Convolution2D	n × m	1	64	56 × 56 × 64
5	Pool 2	MaxPool2D	2 × 2	2	–	56 × 56 × 64
6	Conv 3	Convolution2D	n × m	1	32	28 × 28 × 64
7	Pool 3	MaxPool2D	2 × 2	2	–	28 × 28 × 32
8	Conv 4	Convolution2D	n × m	1	32	14 × 14 × 32
9	Pool 4	MaxPool2D	2 × 2	2	–	14 × 14 × 32
10	–	Dropout (0.4)	–	–	–	7 × 7 × 32
11	–	Flatten	–	–	–	7 × 7 × 32
12	–	Dense_1	–	–	–	1568
13	–	Dense_2	–	–	–	500
14		Output	–	–	–	30

**Table 3:** CNN model structure parameters.

Model	Kernel size
CNN-S22	2 × 2
CNN-S23	2 × 3
CNN-S32	3 × 2
CNN-S33	3 × 3
CNN-S55	5 × 5
MSCNN	Conv1: 2 × 2 Conv2: 2 × 3 Conv3: 3 × 2 Conv4: 3 × 3
EMSCNN	Model1: 2 × 2 Model2: 2 × 3 Model3: 3 × 2 Model4: 3 × 3

In the models of LeNet, VGG16, MobileNetV2, ResNet101, EfficientNetB7, and SPP-net the input image size is uniformly set to 112 × 112 × 3, 500 as the output of the dense layer, and the SoftMax is 30 to start model training and verification. For the Darknet53 model, the input image size is set to 112 × 112 × 3, the SoftMax value is 30, and other parameters are default values for training. The results obtained by establishing the models through experiments are shown in Table 4.

**Table 4:** Model classification results.

Model	Top-1 (%)	Top-5 (%)	Time(s)	Epochs(iterations)
LeNet	87.41	97.38	958	30 × 821
VGG16	54.46	82.72	1127	30 × 821
ResNet101	47.21	78.49	1807	30 × 821
MobileNetV2	72.23	92.79	1243	30 × 821
EfficientNetB7	52.84	79.94	3839	30 × 821
Darknet53	64.73	92.14	32,040	100,000
SPP-net	78.42	95.82	1165	30 × 821
CNN-S22	86.94	97.55	1017	30 × 821
CNN-S23	89.26	97.98	1027	30 × 821
CNN-S32	88.24	97.93	1023	30 × 821
CNN-S33	89.46	97.93	1008	30 × 821
CNN-S55	89.46	97.99	1022	30 × 821
MSCNN	89.61	98.19	1017	30 × 821
EMSCNN	91.49	98.70	1856	30 × 821

**Figure 7:**
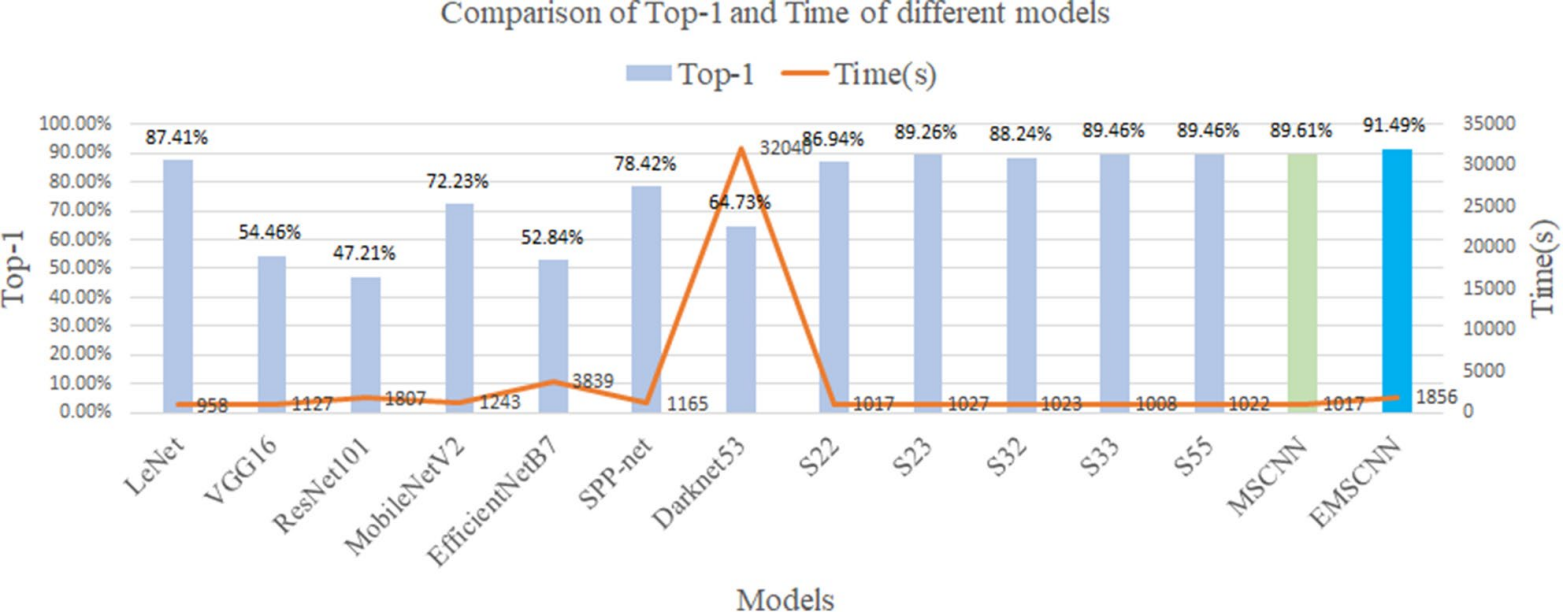
Comparison of Top-1 and Time of different models.

The Top-1, Top-5, model training time, and the number of iterations of the classification model are obtained through experiments, as shown in Table 4. The time of the ensemble model is the sum of the training time of CNN-S22, CNN-S23, CNN-S32, and CNN-S33. The Darknet53 model is run in Visual Studio, OpenCV, and CMake-GUI compilation environments. The default parameters of Darknet53 are selected, and the results of this experiment are obtained by iterating 100,000 times. The remaining model epochs are set to 30 times, and 821 is calculated for each epoch. The Top-1, Top-5 and running time of 13 models can be observed in Table 4. The MSCNN and EMSCNN models proposed in this paper have achieved better results in a limited number of iterations and running time than others. The experiment is described according to the model Top-1 and Time values, as shown in Fig. 7.

**Figure 8:**
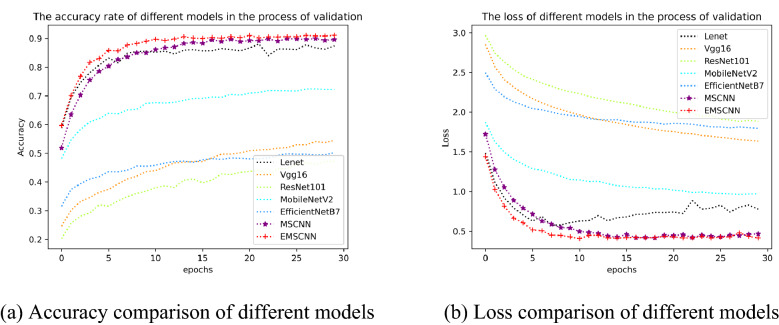
Comparison of MSCNN, EMSCNN and other models in the validation set.

**Figure 9:**
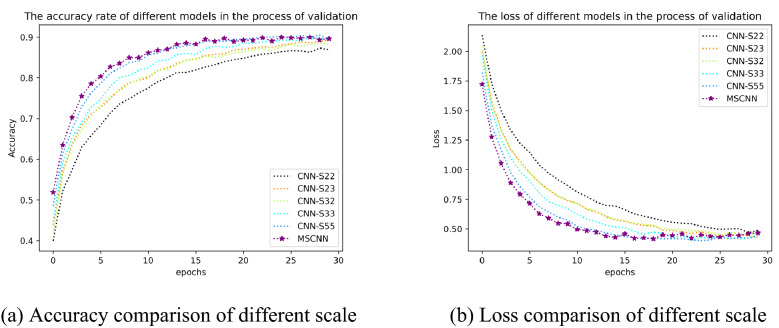
Comparison of MSCNN model results at different scales in the validation set.

**Figure 10:**
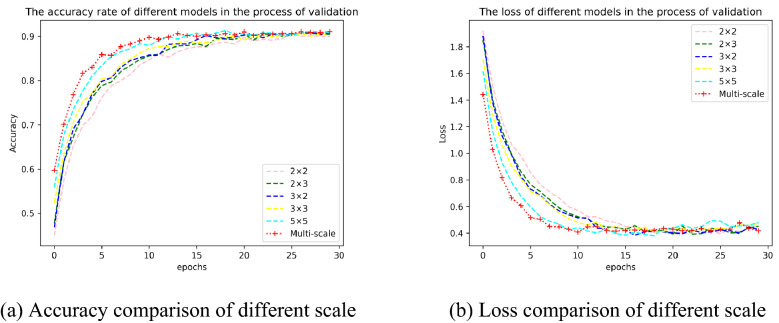
Comparison of EMSCNN model results at different scales in the validation set.

**Figure 11:**
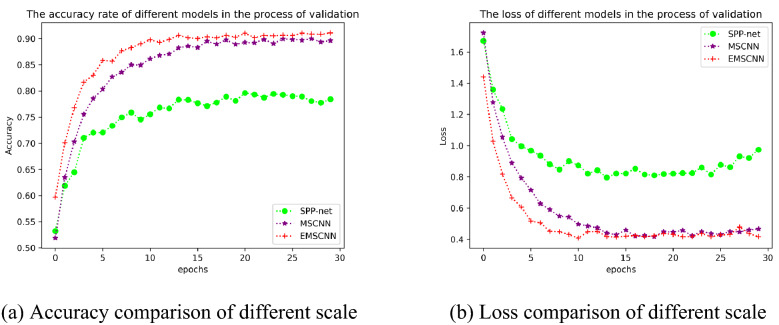
Comparison of MSCNN, EMSCNN and SPP-net in the validation set.

According to the Top-1 comparison of 14 models, it can be seen that our EMSCNN model achieves the best Accuracy compared with other models with the same training time. Compared with other models, our EMSCNN achieves the most outstanding Accuracy with a slight increase in model training time. It shows that our MSCNN and EMSCNN have more significant advantages in efficiency and performance. In order to evaluate the model more comprehensively, the experiment outputs the results of the Accuracy, precision, Recall, F1-score, Accuracy and loss with epochs transformation of the validation model, as shown in Tables 5, 6 and 7 and Figs. 8, 9, 10 and 11.

Table 5 shows the validation of these models: LeNet, VGG16, ResNet101, MobileNetV2, EfficientNetB7, MSCNN and EMSCNN. Our MSCNN and EMSCNN are better than other models and achieve the best results. The accuracy of MSCNN is 2.21%, 35.15%, 42.40%, 17.38% and 36.78% higher than LeNet, VGG16, ResNet101, MobileNetV2 and EfficientNetB7 respectively. The accuracy of EMSCNN is 4.08%, 37.02%, 44.28%, 19.26%, 38.65% and 1.88% outperformance to LeNet, VGG16, ResNet101, MobileNetV2, EfficientNetB7 and MSCNN respectively. The comparison of accuracy and Loss on the validation dataset is shown in Fig. 8.

**Table 5:** Model classification results.

Model	Accuracy (%)	Precision (%)	Recall (%)	F1-score (%)
LeNet^37^	87.41	86.57	86.30	86.27
VGG16^38^	54.46	56.83	51.73	53.20
ResNet101^39^	47.21	51.03	44.30	44.67
MobileNetV2^40^	72.23	74.83	70.03	71.37
EfficientNetB7^41^	52.84	53.70	50.73	51.53
SPP-net^36^	78.42	79.13	76.90	77.57
MSCNN	**89.61**	**89.53**	**88.90**	**88.90**
EMSCNN	**91.49**	**90.73**	**90.07**	**90.30**

The curves in Fig. 8a show the MSCNN and EMSCNN models perform better on the validation dataset; the accuracy curves are more stable and higher than other models with better convergence. The loss curves in Fig. 8b shows the loss of the MSCNN and EMCNN are also relatively stable, and their loss value are lower than other models and converge better.

### Model ablation

To further study the utility of our proposed models, two schemes are designed to verify the performance of MSCNN and EMSCNN, respectively.

#### Different scales of MSCNN model

The classification results of CNN-S22, CNN-S23, CNN-S32, CNN-S33, CNN-S55 and MSCNN models in the validation set are shown in Table 6. The results show that MSCNN achieves quite competitive results on the validation set, and all indicators are higher than other scale CNN models. In Table 6 the accuracy of MSCNN is 89.61%, which is 2.68%, 0.35%, 1.37%, 0.15%, 0.15% higher than CNN-S22, CNN-S23, CNN-S32, CNN-S33, and CNN-S55, respectively. The more details of comparison at different scales model and MSCNN are shown in Fig. 9.

**Table 6:** Classification results of MSCNN at different scales.

Model	Accuracy (%)	Precision (%)	Recall (%)	F1-score (%)
CNN-S22	86.94	86.97	85.73	86.17
CNN-S23	89.26	89.00	88.23	88.60
CNN-S32	88.24	89.00	88.23	88.60
CNN-S33	89.46	88.93	88.37	88.43
CNN-S55	89.46	89.50	88.50	88.83
MSCNN	**89.61**	**89.53**	**88.90**	**88.90**

In Fig. 9(a), the accuracy of MSCNN is higher in the most epochs, and the fluctuation is slight. In Fig. 9(b), the loss of the MSCNN converge faster and has minor changes. Experimental results demonstrate that the multi-scale CNN model can achieve better classification results, and the model performance is more stable, which is helpful for practical application.

#### Different scales of EMSCNN model

In order to further study the difference of the integrated multi-scale model EMSCNN, each model of EMSCNN is built with the single-scale convolution kernel (2 × 2, 2 × 3, 3 × 2, 3 × 3, 5 × 5) while keeping the CNN structure and parameters unchanged. The results are shown in Table 7.

**Table 7:** Classification results of EMSCNN at different scales.

Model	Scale	Accuracy (%)	Precision (%)	Recall (%)	F1-score (%)
EMSCNN	2 × 2	89.93	89.87	88.83	89.10
	2 × 3	90.48	90.33	89.50	89.80
	3 × 2	90.72	90.43	89.97	90.07
	3 × 3	90.17	89.70	89.67	89.53
	5 × 5	90.84	90.57	89.99	90.17
	**Multi-scale**	**91.49**	**90.73**	**90.07**	**90.30**

According to the experimental results, EMSCNN (with multi-scale) proposed in this paper achieves the best results in the different scales. In Table 7 the accuracy of EMSCNN with multi-scale is 91.49%, which is 1.56%, 1.01%, 0.77%, 1.32%, 0.65% higher than the 2 × 2, 2 × 3, 3 × 2, 3 × 3 and 5 × 5 scale of EMSCNN models, respectively. The accuracy of the models on the validation set and the comparative analysis of the change of Loss with epoch are shown in Fig. 10. It can be seen that the multi-scale convolution kernel EMSCNN model can converge quickly, and can obtain better accuracy.

### Discussion

In this paper, we consistently demonstrate that multi-scale CNN models outperform other models for learning wavelet-transformed spectrograms, especially when ensemble multi-scale applications.

With respect to the recognition of speech and birdsong, many researchers often learn multi-scale features directly from waveforms^42–44^ or use short-time Fourier transforms^19,45^, and Mel filters^21,46^ to generate spectrograms as input into CNN. Mel filtering is designed to imitate human hearing habits, and there is a lack of evidence about whether birds have the same characteristics. The method of directly extracting multi-scale features from birdsong waveforms has limited feature scales, and uses a fixed scale of STFT to extract a single feature. The above methods are difficult to adapt the fast-changing frequency of birdsong in a short period of time. The wavelet transform for multi-resolution analysis can effectively overcome these shortcomings. Continuous wavelet transform generates more discriminative multi-scale spectrograms for subsequent convolutions. Secondly, considering the different sensitivity of the convolution kernel scale to the spectrogram, the small-scale convolution kernel is used to extract high-frequency information, and the large-scale convolution kernel extracts low-frequency information. So as shown in Fig. 2, multi-scale convolution kernels are explored to build MSCNN and EMSCNN models.

Recently, CNN has received more attention from researchers in various fields. The structures CNN have shown great potential in classification problems as well as other tasks such as object detection, semantic segmentation, natural language processing. The well-known architectures such as LeNet, VGG16, MobileNetV2, ResNet101, and EfficientNetB7 have become more popular in image classification. Few people have built multi-scale CNN model with WT spectrum for birdsong recognition. This study explored the characteristic of WT of birdsong and multi-scale CNN to propose the MSCNN and EMSCNN architectures. Compared with the performance of LeNet, VGG16, MobileNetV2, ResNet101, and EfficientNetB7, our MSCNN model accuracy improved 2.21–42.4%, EMSCNN model achieves an increase of 2.21–44.28% compared to other models.

Similar to the multi-scale model proposed in this paper, the SPP-net^36^ model achieves better performance in the classification field. SPP-net trains a deep network with a spatial pyramid pooling layer. It can deal with different size of input images. Features extracted at any scale can be pooled. Pyramid pooling makes the network more robust. SPP-net has been applied to object detection, image classification and other fields. In order to better reflect the performance of the model proposed in this paper, an image multi-scale model SPP-net was built in the experiment, and trained on the data set used in this paper. The results are shown in Table 5 and Fig. 11.

The performance of MSCNN and EMSCNN proposed in this paper are better than SPP-net. The accuracy of SPP-net is 78.42%, which is 11.19%, 13.07% lower than MSCNN and EMSCNN, respectively. Experimental results demonstrate the effectiveness of the model proposed in this paper, which may provide a reference for the establishment of subsequent multi-scale models.

However, the method proposed in this paper still has some limitations. First of all, this paper only uses the wavelet transform extraction method to generate the bird song spectrogram, and does not use other feature extraction methods. Second, the proposed network has only been tested on 30 kinds of bird song data, and it is uncertain whether it will be effective in the increasingly complex birdsong data. Third, the division method of the convolution kernel may not be the optimal solution, and further exploration is needed.

## Conclusion

Based on the WT spectrogram, this paper proposed a classification method and explored MSCNN and EMSCNN to solve the problem of birdsong classification. We first generated the WT spectrograms of 30 species birdsongs. The MSCNN and EMSCNN classification models were constructed on the WT spectrogram. The results show that in the 5 × 5 convolution kernel decomposition experiment, the performance of the MSCNN model is better than that of LeNet, VGG16, ResNet101, MobileNetV2, EfficientNetB7, Darknet53 and SPP-net models. The accuracy rate of EMSCNN is more excellent than MSCNN with an increase of 1.88%. In the experiments on 30 bird species, MSCNN and EMSCNN effectively improved the classification effect of the model while ensuring the stability and efficiency of the model compared with other models. All indicators are higher than other models, indicating that the models proposed in this paper have better generalization ability. In the future, we will fuse multi-view birdsong features to explore the applicability of the proposed network, and extend the MSCNN and EMSCNN models to more bird song audios and other audio data classification tasks.
